# Sociodemographic and Health Indicators of Diet Quality in Pre-Frail Older Adults in New Zealand

**DOI:** 10.3390/nu15204416

**Published:** 2023-10-18

**Authors:** Esther Tay, Daniel Barnett, Maisie Rowland, Ngaire Kerse, Richard Edlin, Debra L. Waters, Martin Connolly, Avinesh Pillai, Evelingi Tupou, Ruth Teh

**Affiliations:** 1 Department of General Practice and Primary Health Care, School of Population Health, University of Auckland, Auckland 1023, New Zealand; tseesther@hotmail.com (E.T.); n.kerse@auckland.ac.nz (N.K.); e.leilua@auckland.ac.nz (E.T.); 2 Department of Statistics, Faculty of Science, University of Auckland, Auckland 1010, New Zealand; dbar344@aucklanduni.ac.nz (D.B.); a.pillai@auckland.ac.nz (A.P.); 3 Human Nutrition Research Centre, Population Health Sciences Institute, Faculty of Medical Sciences, Newcastle University, Newcastle Upon Tyne NE2 4HH, UK; maisierowlandrnutr@hotmail.com; 4 Health Systems Group, School of Population Health, University of Auckland, Auckland 1023, New Zealand; r.edlin@auckland.ac.nz; 5 Department of Medicine, University of Otago, Dunedin 9016, New Zealand; debra.waters@otago.ac.nz; 6 Centre for Health, Activity and Rehabilitation Research, School of Physiotherapy, University of Otago, Dunedin 9016, New Zealand; 7 Department of Internal Medicine, University of New Mexico, Albuquerque, NM 87106, USA; 8 Department of Geriatric Medicine, Faculty of Medical and Health Sciences, University of Auckland, Auckland 1023, New Zealand; martinconnolly1957@gmail.com

**Keywords:** frailty, older adults, diet quality, sociodemographic, BMI

## Abstract

This study aimed to identify sociodemographic and health indicators of diet quality in pre-frail community-dwelling older adults. Pre-frail older adults are those at risk of progression to clinical manifestations of frailty and are targets for preventative intervention. We previously reported that pre-frail older adults have reasonably good overall diet quality. However, further analyses found a low intake of energy, protein and several micronutrients. Methods: We collected detailed dietary intake from pre-frail (FRAIL scale 1–2) older adults using NZ Intake24, an online version of 24 h multiple pass dietary recall. Diet quality was ascertained with the Diet Quality Index-International (DQI-I). We used regression generalized linear models to determine predictors of diet quality as well as classification and regression tree (CART) analysis to examine the complex relationships between predictors and identified profiles of sub-groups of older adults that predict diet quality. Results: The median age in this sample (*n* = 468) was 80.0 years (77.0–84.0). Living with others, a high deprivation index and a higher BMI were independent predictors of poorer diet quality. With CART analysis, we found that those with a BMI > 29 kg/m^2^, living with others and younger than 80 years were likely to have a lower diet quality. Conclusions: We found that BMI, living arrangement and socioeconomic status were independent predictors of diet quality in pre-frail older adults, with BMI being the most important variable in this sample when the interaction of these variables was considered. Future research is needed to determine the similarities and/or differences in the profile of subgroups of older adults with poorer diet quality.

## 1. Introduction

Frailty is a condition of accumulated decline in physiological reserves resulting in weakened homeostatic responses to stressors [1,2]. The prevalence of physical frailty increases with age, with almost half of individuals aged over 50 years old classified as pre-frail and 12–24% as frail [3]. In a New Zealand survey, Māori living in the community had a 10-to-15 years earlier onset of frailty compared to non-Māori [4]. Older adults displaying features of frailty are vulnerable to poor health outcomes like falls and prolonged hospital stays [1]. Pressure is put on the health sector to work against this inequity and reduce the burden of frailty in the community, especially population groups most at risk.

Poor nutrition can influence frailty indicators such as weight loss and exhaustion [1]. A systematic review and meta-analysis of 10 studies (*n* = 5447 community-dwelling older adults, mean age 77 years) found that one in four malnourished older adults were pre-frail, and two-thirds were frail [5]. Increasingly, studies have also found higher risks of frailty in older adults with obesity and sarcopenic obesity (low muscle mass and function coupled with high fat mass) [6,7,8,9]. In the NHANES study, pre-frail older adults had greater central adiposity than their robust counterparts [7]. These findings suggest that, even at early stages of frailty progression, or else termed “pre-frailty” [10], both undernutrition and overnutrition may be key risk factors (amongst many others).

Diet quality is a measure of the quality, quantity and variety of the entire diet, allowing for the examination of the association between whole food and health status [11]. Many factors are associated with diet quality in older adults. The complexity of each population group and diet quality determinants make it challenging to single out individual factors as, collectively, there are many factors to consider regarding varying interactions dependent on specific situations [12]. Sociodemographic and health indicators are associated with diet quality in older adults [13,14,15,16,17,18,19,20,21,22,23,24,25,26,27]. In frail older adults, the higher presence of mobility and functional limitations and poorer health status may impact access to healthy food, predisposing them to the risk of malnutrition [1,5,28].

We have previously described the diet quality of pre-frail older adults in New Zealand [29]. We found that pre-frail older adults had nutritional gaps in energy and nutrient deficiencies and a disproportionate consumption of empty calories. Dietary moderation and balance scores averaged below 50% of the maximum score in the diet quality index–international (DQI-I) tool [29]. The nutritional gaps call into question how clinicians and policymakers can influence diet quality in at-risk groups for frailty prevention. This current paper aimed to identify the predictors of diet quality in pre-frail community-dwelling older adults and identify mutually exclusive subgroups in the sample who shared common characteristics that influence diet quality.

## 2. Materials and Methods

### 2.1. Study Sample

Community-dwelling older adults aged 75+ (or 60 for Māori and Pacific people) were recruited across four sites in New Zealand (Auckland, Whangarei, Tauranga and Invercargill) through general practices via postal invitations to participate in the Staying UPright and Eating well Research (SUPER) study. Those who were terminally ill, had advanced dementia or for whom it was medically unsafe to participate in low-intensity exercise as judged by the GP or Māori health provider were not invited to the study.

Those who responded with interest (4791 of 6690 invited) were screened for pre-frailty using the self-administered FRAIL questionnaire. Those scoring 1 or 2 out of 5 in the tool (i.e., pre-frailty) and self-reported being able to stand and able to use the kitchen utensils safely were recruited [30]. Of those screened, 1015 were eligible and 468 pre-frail older adults were recruited. The lower threshold for Māori and Pacific people was implemented to reflect the inequitable earlier occurrence of disability, poor health and mortality in these population groups compared to the general population in New Zealand [31]. All study participants provided written informed consent.

The Southern Health and Disability Ethics Committee, Ministry of Health, New Zealand (Ref 14/STH/101/, 13 August 2014) approved this study.

We utilized the baseline data of the SUPER Study collected between 2016 and 2018 for this analysis.

### 2.2. Data Collection

Dietary intake was recorded on two separate days using a 24 h multiple pass dietary recall method (24 h-MPR) through the online Intake24 platform developed by Newcastle University [32,33]. The 24 h-MPR has proved to be a feasible dietary assessment method to capture detailed dietary intake in older adults. [34] We adapted the Intake24 online platform to be relevant to New Zealand older adults’ dietary intake based on the 24 h-MPR paper-based version from more than 500 octogenarians. The backend of the Intake24 online platform was updated with the New Zealand FOODfiles database from 2014 to 2016 [35], leading to 158 food codes being withdrawn and 215 updated. Meticulous data verification and coding were completed by qualified nutritionists, ensuring the database fit the purpose of the study. A workflow summary is presented in the supplementary material (Figure S1).

Diet quality was ascertained using the Diet Quality Index-International (DQI-I) tool developed by Kim et al., which measures intake according to four categories: variety (20 points), adequacy (40 points), moderation (30 points) and balance (10 points)—that adds up to a maximum score of 100; a higher score indicates a better diet quality [36]. The DQI-I is validated for international comparisons and has previously been utilized effectively in older population groups [29,37]. Adaptations to the DQI-I for our study were reported previously [29].

Sociodemographic and health information collected using a standardized questionnaire and at-home interviews included: age, sex, ethnic group, education level, New Zealand Deprivation Index, living arrangement, medical conditions, medications, supplements, alcohol, smoking, vision, hearing, Nottingham Extended Activities of Daily Living (NEADL), Montreal Cognitive Assessment (MoCA), Geriatric Depression Scale (GDS) and short physical performance battery (SPPB) [30].

Anthropometric information was measured using a Tanita BC-545N scale and Stadiometer. BMI was calculated as weight (kg)/height (m)^2^. Waist circumference was measured with a non-stretchable tape taken at the natural narrowing midway between the last rib and the crest of the ilium.

Further data collection details are recorded in the study protocol [30].

### 2.3. Statistical Tests

#### 2.3.1. Descriptive Data

Quantitative data are presented as median (interquartile range (IQR)) or mean (standard deviation (SD)) depending on data distribution, and categorical data are presented as count (percentage).

#### 2.3.2. Regression and Tree Analysis

Generalized linear models (GLMs), with normal probability distribution and identity link function, were constructed to identify predictors of DQI-I scores. All independent variables (listed in Table 1) except BMI, waist circumference, NEADL and SPPB were categorical. Variables with a *p*-value less than 0.2 from univariate models were systematically included in the multivariate GLM models to construct parsimonious models. To select a subset of predictors for the models, we added potential predictors of DQI-I scores by groups, i.e., first, we included sociodemographic variables, followed by lifestyle variables (smoking, alcohol), sensory (hearing, vision), body composition (BMI, waist circumference), physical function (NEADL, SPPB) and health status (co-morbidity, prescribed medication, supplement and MoCA). After selecting a subset of predictor variables, we constructed parsimonious models using Akaike Information Criterion and Bayesian Information Criterion. We presented the parsimonious models (Table 2). Further analyses were conducted on DQI-I subcomponents, i.e., diet variety, adequacy, moderation and overall balance.

Classification and regression tree (CART) analysis is a machine learning technique that helps make predictions by untangling interactions between variables and splitting data into subgroups based on a criterion that aims to maximize the distinction of the outcome of interest through a recursive process to produce mutually exclusive and exhaustive subgroups of population that share common characteristics that predict the outcome of interest [38]. We used CART to identify mutually exclusive sub-groups within our sample by partitioning groups based on the interaction between demographic and health characteristics that best describe diet quality. The minimum number of cases for child nodes was set to five (1% of the total sample) [38], and the maximum number of tree depths was 5. A k-fold cross-validation was used to improve confidence in the final trees. We choose *k* = 10, a commonly used value which divides the dataset into ten equal-sized subsamples, and performed ten iterations of model training and testing. In each iteration, one subsample of data was reserved as the test set, while the remaining nine subsamples were used for training. After all ten iterations were completed, a single final tree model was produced. Separate CART analyses were completed for low-energy reporters (LER = total energy intake/basal metabolic rate ≤ 0.92) and plausible reporters due to the strong relationship between low-energy reporting and DQI-I [29]. We then compared the results obtained from CART with the GLM regression models.

Statistical significance was set at *p* < 0.05. All statistical tests in this paper were conducted using IBM SPSS Statistics for Macintosh, version 27 (IBM Corp., Armonk, NY, USA).

## 3. Results

### 3.1. Sample Descriptives

Of the sample of 468 participants recruited from 27 general practices, three had missing dietary assessments due to technical difficulties. The median age was 80.0 years (77.0–84.0), 59% were female and a majority were of European descent (Table 1).

### 3.2. Associations: Generalized Linear Models

#### 3.2.1. Univariate Analysis

In univariate analysis, living alone, low deprivation and lower BMI were significantly associated with higher (better) overall DQI-I score (*p* < 0.05) (Table A1). More specifically, those living alone had a higher score for moderation of food/nutrient intake that may require restriction, e.g., saturated fat and sodium; lower BMI was associated with higher scores in variety and adequacy, and better socioeconomic status was associated with higher adequacy scores.

Subgroup analyses were completed for low-energy reporters (*n* = 151, 32.5%, Table A2) and plausible reporters (*n* = 313, 67.5%, Table A3). Among plausible reporters, non-European participants had a lower diet quality score than their European counterparts, attributable to a lower adequacy score. 

#### 3.2.2. Multivariate Analysis

Multivariate analyses show that those living with others (*p* = 0.042), with lower socioeconomic status (*p* = 0.024) and a higher BMI (*p* = 0.026) were associated with poorer diet quality (lower DQI-I scores) (Table 2). Living with others and having a higher BMI were associated with lower diet variety scores. Lower socioeconomic status and higher BMI were associated with lower adequacy scores. We observed that impaired cognitive function and regular alcohol consumption were associated with overall balance in dietary score (Table 2). 

In subgroup analyses completed among LER, we found no associations between demographic and health variables with DQI-I. In further analyses, we observed various variables associated with adequacy, moderation and balance. Participants living in medium-deprived areas were associated with higher adequacy than those living in highly deprived areas (*p* = 0.007); the difference was not seen between low and high areas (*p* = 0.111) (Table S1). For plausible reporters, associations were found for DQI-I and all subcomponents apart from variety. Participants of non-European ethnicity were marginally associated with a lower DQI-I, which is linked to a lower adequacy score (*p* = 0.003). Smokers were associated with lower adequacy (*p* = 0.027) than non-smokers (Table S2).

### 3.3. Relationships between Variables: Classification and Regression Tree (CART) Analysis

**Figure 1:**
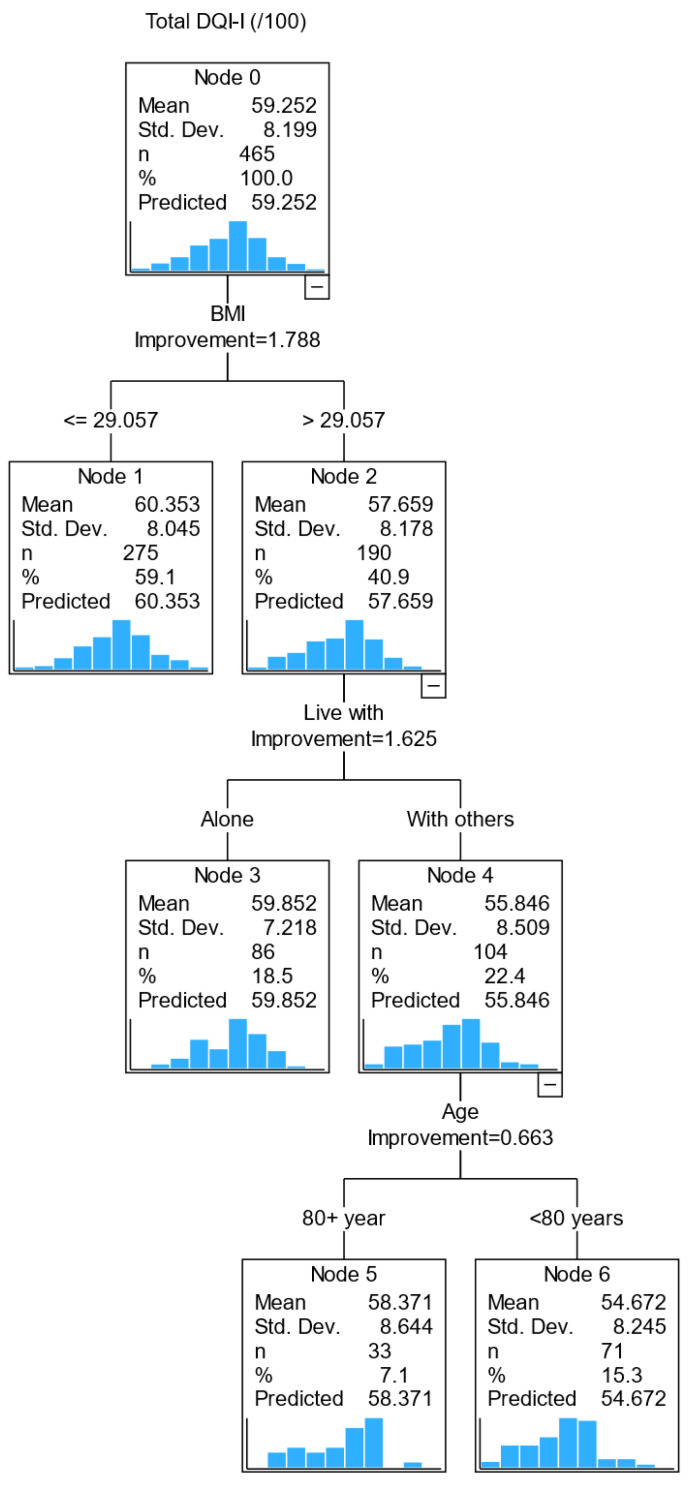
CART diagram of factors associated with diet quality (DQI-I) in pre-frail older adults.

We completed a regression tree analysis to identify the profile of subgroups of older adults with poorer diet quality. Variables with a relative importance value above 90% in predicting diet quality were age (100%), BMI (99.2%) and living arrangement (92.7%), followed by vision (85.4%), and each of the remaining variables had a relative importance value less than 50%. In the decision tree model below, splitting the sample into mutually exclusive subgroups (Figure 1), only BMI, living arrangement and age were included; vision was excluded from the final tree model to prioritize more stable predictors.

The first decision point is whether an older adult has a BMI above or below 29 kg/m^2^ (i.e., the best-split value from the regression tree analysis). A person with a BMI of >29 kg/m^2^ was predicted to have a lower diet quality than those with a BMI of ≤29 kg/m^2^. The second decision point was the living arrangement, i.e., living with others or alone. Those with a BMI > 29 kg/m^2^ and living with others had a lower DQI score than those who lived alone with a similar BMI range. The third decision point was age (<80 and ≥80). Those with a BMI > 29 kg/m^2^, living with others and less than 80 years old had a lower diet quality than those aged ≥ 80. This model identified that those with a BMI > 29 kg/m^2^ living with others younger than 80 years were likely to have a lower diet quality.

Separate tree diagrams were constructed for DQI-I subcomponents (Figure S1). In each tree diagram, BMI remains a key (first) independent variable for all subcomponents, except for moderation, where sex was a predictor of moderation score (male sex has a lower moderation score). The second independent variable varies across the DQI-I subcomponents: living arrangement for diet variety, medication for adequacy and NEADL for balance.

When we compared the tree diagrams (CART analysis) to the GLM regression models, we found BMI to be a consistent predictor for the overall DQI-I score and subcomponents’ adequacy and balance scores; sex was also consistently identified for the moderation score in both the GLM model and the CART analysis.

## 4. Discussion

This paper aims to identify predictors of diet quality in community-dwelling pre-frail older adults and to identify mutually exclusive subgroups in the sample who share common characteristics that influence diet quality. We found that BMI, living arrangement and socioeconomic status were associated with diet quality. In GLM models, those with low socioeconomic status, living with others and higher BMI were independently associated with poorer diet quality.

We observed that a higher BMI was independently associated with lower diet quality, and further analysis showed a similar association for subcomponent diet adequacy. Diet adequacy was ascertained from vegetable, fruit and grain food groups, as well as key nutrients protein, iron, calcium and vitamin C [36]. Socio-economic status is linked to accessibility to food. A systematic review reports that socio-economic status is closely related to food insecurity in older adults, which impacts nutritional status [39]. A severe level of food insecurity characterized by food scarcity and hunger leads to weight loss, while a moderate level of food security characterized by scarcity of nutritional quality food leads to overweight [39]. In our study sample, those living in an area with a high deprivation index had a poorer diet quality.

It is somewhat surprising that living alone was independently associated with better diet quality, contrary to a previous study on octogenarians in which living alone was associated with high nutrition risk [40]; in a younger group of adults (mean age 74 ± 12), there was no conclusive evidence on the link between living arrangement and nutrition risk [41]. This is likely to be attributed to the interplay between living arrangement and other factors; for example, social networks in the neighborhood were shown to attenuate the negative association between living alone and daily fruit and vegetable consumption [42]. Two studies reported that some older adults living on their own relished the opportunity to experiment with different foods while others felt a sense of release from the constraint of eating food that pleased the preferences of others [16,43]. These findings may partly explain the higher diet quality score of those living alone in our study. We recommend further research to untangle the interaction of social networks and diet quality in older adults.

Further analyses into the subcomponents of diet quality found several interesting observations. Firstly, we observed that alcohol intake was associated with diet variety and balance scores. In the NHNES survey on a younger population, total diet quality (measured using the Healthy Eating Index) increased with the frequency of alcohol intake [44]. In our sample, there was no association between diet quality and alcohol consumption. However, those who never had alcohol had a lower diet variety and a higher dietary balance score than those who reported having alcohol more regularly. A lifestyle behavior (no alcohol or supplement) may be a key factor to a better dietary balance score. Another mechanism in play could be a compensating mechanism between diet variety and balance among non-alcohol users; the impact of this compensation over time on physical frailty needs further investigation.

Secondly, we did not find an independent association between smoking status and diet quality, but we observed that non-smokers had better diet adequacy and variety scores than smokers. These are in line with previous findings [45,46]. In the NHNES study, dietary energy density, a marker of diet quality, was associated with smoking status [45]. The authors found that smokers had a significantly higher dietary energy density (associated with poorer diet) than never-smokers [45]. The SRISCAV-LUX study (a younger population age 18–69, mean age approximately 42) reported that cigarette smoking was inversely associated with overall diet quality [46]. Although we did not observe a significant association between smoking status and diet quality, our findings showed a similar trend, as reported previously; the small sample size may have contributed to type II error.

Thirdly, we observed that those who used two or more medications had a lower diet adequacy score than those who used one or none. A similar trend was observed among those with and without cognitive impairment, i.e., the former group had a better dietary balance score. A Canadian study also observed this paradoxical relationship between the number of medications and cognitive function. [47] Reverse causality may contribute to this observation. In the NuAge study, total diet quality was not independently associated with cognition in healthy older adults (mean age 74). [47] The authors postulate that other risk factors may moderate the relationship between diet quality and cognition. Evidence on the effect of single nutrient supplementation on cognitive impairment is inconclusive, and a Mediterranean diet may help reduce the risk of cognitive decline [48].

A final observation on the subcomponents of diet quality was that the male sex was associated with a lower diet moderation score than its female counterpart. We reported previously that sodium intake was higher in males (median (interquartile range): 2140 (1701–2800) mg/day) than in females (1595 (1251–2160) mg/d); this is likely to contribute to a lower moderation score observed in men [29]. When all subcomponents were considered, sex was not an independent predictor of total diet quality in our sample of pre-frail older adults, a finding which is different from previous studies in younger or healthy populations [20,47]. Our findings of a more in-depth insight into predictors of specific aspects of diet quality could be useful for more targeted dietary interventions to impact physical frailty.

Findings from the CART analysis help to untangle the interaction of all variables of interest. We observed that a BMI > 29 kg/m^2^ was the best-split value in predicting diet quality, i.e., those with BMI > 29 kg/m^2^ had a lower diet quality (57.6) than those with BMI ≤ 29 kg/m^2^ (60.4). Further subgroups were identified based on living arrangement, i.e., those with BMI > 29 kg/m^2^ and living with others have a lower diet quality score than those who live alone. Further analysis showed that BMI remains the most important factor in predicting all subcomponents of diet quality except for moderation (i.e., energy intake from dietary fat, cholesterol, sodium and empty calorie foods), in which the male sex had a lower moderation score than its female counterpart. After BMI, living arrangement was predictive of diet variety; medication was predictive of diet adequacy and NEADL score for the overall balance of macronutrient and fatty acid ratios.

Previous studies found that diet quality is linked to health outcomes. In a longitudinal study of more than 7000 adults with a mean age of 65 years, diet quality was associated inversely with ADL-based disability and depression [49], and the Physicians Health Study found a dose–response relationship between diet quality with pre-frailty and frailty [50]. Enhancing diet quality can be advantageous for older adults. One approach to enhance diet quality in older adults is increasing nutritional literacy (e.g., interpreting food labels), awareness of food choices and knowledge of healthy food swaps.

These three diagrams make it possible for primary care providers to visualize factors important for dietary quality in pre-frail older adults. Nutrition plays a vital role in physical and mental health. Inadequate nutrition can exacerbate pre-frailty progression to the clinical manifestation of frailty [51]. The early identification of factors important for diet quality may facilitate a timely and specific intervention (e.g., education on increasing diet variety and/or specific nutrients, or moderation in empty calories) to optimize physical functionality.

### Strengths and Limitations

We collected detailed dietary intake from a sample of community-dwelling older adults using the 24 h multiple pass dietary recall method. To the best of our knowledge, this is the first paper to explore the sociodemographic, socioeconomic and health indicators associated with the diet quality of pre-frail older adults.

This study has several limitations, including the nature of the data analysis. We cannot draw a cause–effect relationship from the results observed in this study. Influence can go both ways. Longitudinal studies are warranted to identify the directions and effects of determinants [12]. We were restricted to completing a training validation for the CART analysis to increase confidence and reduce the risk of bias in the study findings to allow for some external validation [52]. However, with the current sample size (*n* = 468), k-fold cross-validation is acceptable [38]. A small sample size is prone to prediction errors, including erroneous splits within the tree structures. In our GLM regression models, BMI and living alone were independent predictors of diet quality, agreeing with CART analysis regarding the strength and direction of the association.

This sample includes only pre-frail older adults, as determined using the FRAIL scale. This limits the generalizability of our findings to those who are “robust” or “frail”. Also, the FRAIL scale is composed of five items: fatigue, resistance, ambulation, number of illnesses and unintentional weight loss greater than 5% (12), and it was self-administered, which may be subject to recall bias (medical conditions) or social desirability bias (ambulation, climbing stairs without aid or physical activity levels). While a more objective measure of frailty phenotype (e.g., Fried phenotype of frailty) is desired, a simple test is desired in a primary care setting where resources are constrained. Similarly, finding the right balance between the availability of resources (e.g., trained clinicians) and the robustness of a tool to detect an outcome of interest need is essential. The FRAIL scale has a moderate-to-good agreement with many published frailty measures, including the Fried phenotype of frailty and Comprehensive Geriatric Assessment (CGA) [53].

The 24 h MPR dietary assessment method is subjected to biases, including social desirability, memory and seasonal variability. However, it minimizes participants’ burden and employing multiple passes allows for detailed information about portion sizes, recipes and preparation methods. This method was used by the New Zealand Adult National Nutrition Survey [54] and validated in advanced age [34,55]. The online platform relies on good network coverage and comprehensiveness of the food composition databases.

Both Māori and Pasifika people are disproportionately represented in poorer health outcomes. Under the Treaty of Waitangi and the goal of reducing inequalities in health, this study was designed to include Māori and Pasifika elders. The study sample included only a small proportion of Māori and Pasifika participants (9%). The proportion of Māori and Pasifika adults aged 60+ in the New Zealand population in 2013 was 6.6% [56] {Statistics New Zealand, #2753}. With the doubling of Māori and Pasifika elders in the next two decades, more robust research with Māori and Pasifika elders is warranted to enhance the understanding of the links between diet quality and frailty.

Future research is needed to identify predictors of diet quality in other settings and beyond pre-frail older adults to determine similarities and/or differences of subgroups of older adults who may benefit from timely and targeted nutrition intervention to improve diet quality.

## 5. Conclusions

Sociodemographic and health factors are associated with dietary intake in older adults. We found that BMI, living arrangement and socioeconomic status were associated with diet quality in pre-frail older adults, and the profile of subgroups with poorer diet quality were identified. The findings from this paper add a different dimension of appropriate screening strategies to improve the nutritional status of older adults at risk of frailty.

## Figures and Tables

**Table 1:** Sample characteristics.

Characteristics	All (*n* = 465)
Age, *n* (% ≥80 years)	242 (52.0)
Sex, *n* (% female)	275 (59.1)
Ethnic group ^a^, *n* (% European/ Pākehā)	421 (90.5)
Education level, *n* (%)	
Primary	14 (3.0)
Secondary	256 (55.1)
Tertiary	195 (41.9)
Living arrangement, *n* (%)	
Alone	204 (43.9)
With others	261 (56.1)
NZ Dep Index 2018, *n* (%)	
Low, 1–4	174 (37.4)
Medium, 5–7	159 (34.2)
High, 8–10	132 (28.4)
Medical conditions, *n* (%)	
0–1	112 (24.1)
≥2	353 (75.9)
Vision, *n* (% impaired)	32 (6.9)
Hearing, *n* (% impaired)	35 (7.5)
BMI (kg/m^2^) median (IQR)	28.3 (25.3–31.4)
Waist circumference (cm) median (IQR)	
Male	102.5 (95.8–109.5)
Female	93.8 (85.4–101.9)
Medications, *n* (%)	
1	46 (9.9)
≥2	419 (90.1)
Supplements, *n* (%)	
0	261 (56.1)
≥1	204 (43.9)
Alcohol consumption, *n* (%)	
Never	111 (23.9)
Occasional	187 (40.2)
Regular	167 (35.9)
Smoking, *n* (% smoker)	5 (1.1)
NEADL score, median (IQR)	20 (18–21)
SPPB score, median (IQR)	9 (8–10)
MoCA, median (IQR)	25 (23–27)
Cognitive impairment (MoCA score ≤22), *n* (%)	100 (21.5)
Total DQI-I scores, median (IQR)	60 (11)
Variety, median (IQR)	14 (5)
Adequacy, median (IQR)	30 (7)
Moderation, median (IQR)	12 (6)
Balance, median (IQR)	7 (2)

Note: missing data for waist circumference (3), BMI (5) and MoCA (1). ^a^ Ethnicity: 39 Māori, 4 Pasifika, 2 Asians and 1 Syrian and South African, respectively. BMI, body mass index; DQI-I, Diet Quality Index-International; IQR, interquartile range; MoCA, Montreal Cognitive Assessment; NEADL, Nottingham Extended Activities of Daily Living; NZ Dep Index, New Zealand Deprivation Index; SPPB, short physical performance battery.

**Table 2:** Multivariate regression against DQI-I score and subcomponents.

Demographic and Health Variables	B	95% CI	*p*-Value
DQI-I total score			
Age (<80 year; reference: 80+ years)	−0.99	−2.55, 0.57	0.213
NZ Dep Index 2018 (low; reference: high)	2.14	0.29, 3.99	*0.024*
(Medium; reference: high)	1.49	−0.39, 3.37	0.121
Living arrangement: Live alone; reference: with others	1.60	0.06, 3.15	*0.042*
BMI, kg/m^2^	−0.17	−0.33, −0.02	*0.026*
Smoking (reference: smoker)	5.91	−1.22, 13.03	0.104
NEADL	64.32	56.53, 73.20	0.164
Variety score			
Deprivation (low; reference: high)	0.38	−0.37, 1.14	0.320
(Medium; reference: high)	0.28	−0.49, 1.04	0.478
BMI, kg/m^2^	−0.06	−0.12, 0.01	0.068
Alcohol consumption (never; reference: regular)	−0.84	−1.65, −0.04	*0.040*
(Occasional; reference: regular)	−0.23	−0.93, 0.47	0.526
Smoking (reference: smoker)	2.71	−0.20, 5.63	0.068
SPPB score	0.13	−0.02, 0.27	*0.086*
Adequacy score			
(Intercept)	26.378	21.657, 31.099	<*0.001*
Deprivation (low; reference: high)	1.08	0.09, 2.07	*0.033*
(Medium; reference: high)	0.97	−0.04, 1.97	0.060
BMI, kg/m^2^	−0.10	−0.181, −0.018	*0.016*
Smoking (non-smoker; reference: smoker)	3.92	0.08, 7.75	*0.045*
Medications (1; reference: 2+)	−1.54	−2.87, −0.20	*0.025*
NEADL	0.15	−0.03, 0.32	0.093
Moderation score			
Age, (<80 years; reference: 80+ years)	−0.65	−1.41, 0.11	0.095
Sex (male; reference: female)	−1.41	−2.18, −0.63	<*0.001*
Balance score			
Deprivation (low; reference: high)	0.28	−0.06, 0.62	0.104
(Medium; reference: high)	0.29	−0.05, 0.64	0.092
Supplement (no, reference: yes)	0.31	0.04, 0.59	*0.023*
Alcohol consumption (never; reference: regular)	0.38	0.02, 0.73	*0.039*
(Occasional; reference: regular)	0.28	−0.02, 0.59	0.072
MoCA score (not impaired; reference: impaired)	−0.49	−0.82, −0.16	*0.003*

Italicized *p*-values are significant at the 0.05 level. Note: B, beta coefficient; BMI, body mass index; CI, confidence interval; MoCA, Montreal Cognitive Assessment; NEADL, Nottingham Extended Activities of Daily Living; NZ Dep Index 2018, New Zealand Deprivation Index 2018; SPPB, short physical performance battery.

## Data Availability

The data presented in this study are available upon request from the corresponding author.

## Supplementary Materials

The following are available online at https://www.mdpi.com/article/10.3390/nu15204416/s1, Figure S1: Summary of systems and changes implemented for cleaning Intake24 data, Figure S2: CART diagram of factors associated with DQI-I subcomponents in pre-frail older adults, Figure S3: CART diagram of factors associated with DQI-I and subcomponents in pre-frail older adults for LER only. Figure S4: CART diagram of factors associated with DQI-I and subcomponents in pre-frail older adults for low energy reporter (LER) only. Table S1: Multivariate regression against DQI-I score and subcomponents for Low Energy Reporter (LER) only, Table S2: Multivariate regression against DQI-I score and subcomponents for plausible reporters only.

## Appendix A

**Table A1:** Univariate characteristics associated with DQI-I and subcomponents.

Demographic and Health Variables	DQI-I			Variety			Adequacy			Moderation			Balance		
	B	Std. Error	*p*-Value	B	Std. Error	*p*-Value	B	Std. Error	*p*-Value	B	Std. Error	*p*-Value	B	Std. Error	*p*-Value
Age, (<80 years; reference: 80+ years)	−1.474	0.7572	0.052	−0.247	0.3085	0.423	−0.426	0.4101	0.299	−0.622	0.3931	0.114	−0.178	0.1395	0.202
Sex (male; reference: female)	−0.776	0.7718	0.314	0.375	0.3132	0.231	0.083	0.4173	0.843	−1.394	0.3953	<*0.001*	0.159	0.1418	0.261
Ethnic group (European; reference: non-European)	2.260	1.2934	0.081	0.458	0.5264	0.384	1.381	0.6979	*0.048*	0.117	0.6727	0.862	0.305	0.2381	0.200
Education level (primary; reference: tertiary)	0.041	2.2660	0.985	−0.108	0.9196	0.907	−1.280	1.2224	0.295	1.201	1.1728	0.306	0.227	0.4164	0.585
(Secondary; reference: tertiary)	−0.079	0.7784	0.919	−0.225	0.3159	0.477	−0.205	0.4199	0.625	0.365	0.4029	0.365	−0.014	0.1430	0.924
Live with (alone; reference: others)	1.578	0.7619	*0.038*	0.356	0.3103	0.251	0.341	0.4131	0.409	0.999	0.3941	*0.011*	−0.118	0.1406	0.399
Deprivation (reference: high) Low	1.959	0.9405	*0.037*	0.604	0.3827	0.114	1.137	0.5072	*0.025*	−0.082	0.4900	0.867	0.300	0.1731	0.083
Medium	1.621	0.9595	0.091	0.474	0.3904	0.225	1.112	0.5174	*0.032*	−0.244	0.4998	0.626	0.279	0.1766	0.115
Medical conditions (1; reference: 2+)	0.120	0.8882	0.892	−0.004	0.3606	0.992	−0.244	0.4796	0.611	0.129	0.4604	0.779	0.238	0.1629	0.143
Vision (impaired; reference: non impaired)	−0.569	1.5001	0.705	−0.546	0.6086	0.370	0.513	0.8100	0.527	−0.108	0.7778	0.890	−0.428	0.2751	0.120
Hearing (impaired; reference: non impaired)	1.966	1.4367	0.171	−0.008	0.5845	0.989	1.046	0.7760	0.178	0.950	0.7450	0.202	−0.022	0.2646	0.934
BMI, kg/m^2^	−0.194	0.0769	*0.012*	−0.062	0.0312	*0.047*	−0.093	0.0416	*0.025*	−0.023	0.0401	0.574	−0.016	0.0141	0.258
Waist circumference, cm	−0.055	0.0298	0.064	−0.009	0.0121	0.461	−0.022	0.0160	0.165	−0.022	0.0155	0.150	−0.002	0.0055	0.753
Medications (1; reference: 2+)	−1.160	1.2710	0.361	0.403	0.5162	0.435	−1.236	0.6847	0.071	−0.248	0.6594	0.707	−0.079	0.2338	0.735
Supplements (0; reference: 1+)	−0.133	0.7653	0.862	−0.274	0.3105	0.377	−0.278	0.4132	0.501	0.106	0.3968	0.790	0.314	0.1399	*0.025*
Alcohol consumption (never; reference: regular)	−0.369	1.0012	0.713	−0.806	0.4055	*0.047*	−0.311	0.5409	0.565	0.428	0.5192	0.410	0.321	0.1835	0.081
(Occasional; reference: regular)	0.806	0.8704	0.355	−0.289	0.3525	0.412	0.316	0.4702	0.501	0.501	0.4514	0.267	0.278	0.1596	0.082
Smoking (non-smoker; reference: smoker)	4.936	3.6754	0.179	2.202	1.4917	0.140	3.936	1.9806	*0.047*	−2.404	1.9059	0.207	1.202	0.6746	0.075
NEADL score	0.219	0.1662	0.188	0.052	0.0676	0.444	0.143	0.0897	0.112	0.042	0.0863	0.623	−0.018	0.0306	0.562
SPPB score	0.187	0.1785	0.295	0.172	0.0721	*0.017*	0.123	0.0963	0.202	−0.099	0.0925	0.286	−0.009	0.0328	0.781
MoCA score (not impaired; reference: impaired)	−0.507	0.9252	0.583	−0.307	0.3752	0.414	−0.413	0.4986	0.408	0.700	0.4779	0.143	−0.488	0.1686	*0.004*
GDS score (no depression; reference: depression)	−0.228	1.0013	0.820	−0.124	0.4065	0.760	0.028	0.5409	0.959	−0.313	0.5190	0.546	0.181	0.1839	0.324

Italicized *p*-values are significant at the 0.05 level.

**Table A2:** Univariate characteristics associated with DQI-I and subcomponents for LER only.

Demographic and Health Variables	DQI-I			Variety			Adequacy			Moderation			Balance		
	B	Std. Error	*p*-Value	B	Std. Error	*p*-Value	B	Std. Error	*p*-Value	B	Std. Error	*p*-Value	B	Std. Error	*p*-Value
Age, (<80 years; reference: 80+ years)	−1.838	1.3889	0.186	−0.344	0.5442	0.527	−0.754	0.7696	0.327	−0.600	0.6749	0.374	−1.404	2.4930	0.573
Sex (male; reference: female)	−0.732	1.4186	0.606	−0.112	0.5537	0.840	0.697	0.7826	0.373	−1.763	0.6726	*0.009*	4.461	2.5103	0.076
Ethnic group (European; reference: non-European)	−0.733	2.4075	0.761	0.019	0.9393	0.983	0.098	1.3309	0.941	−1.502	1.1601	0.195	6.520	4.2694	0.127
Education level (primary; reference: tertiary)	0.734	4.4166	0.868	1.427	1.7175	0.406	0.094	2.4257	0.969	0.542	2.1298	0.799	−13.295	7.8215	0.089
(Secondary; reference: tertiary)	−0.736	1.4238	0.605	−0.325	0.5537	0.557	−1.146	0.7820	0.143	0.896	0.6866	0.192	−1.615	2.5214	0.522
Live with (alone; reference: others)	2.731	1.4060	0.052	1.038	0.5488	0.058	1.034	0.7822	0.186	0.956	0.6851	0.163	−2.985	2.5313	0.238
Deprivation (low; reference: high)	2.422	1.7735	0.172	0.049	0.6975	0.943	1.910	0.9638	*0.047*	0.030	0.8638	0.972	4.318	3.1674	0.173
(Medium; reference: high)	2.507	1.7245	0.146	0.106	0.6782	0.876	2.570	0.9372	*0.006*	−0.630	0.8400	0.453	4.612	3.0799	0.134
Medical conditions (1; reference: 2+)	−1.263	1.5795	0.424	−0.183	0.6172	0.767	−0.657	0.8731	0.452	−0.709	0.7646	0.354	2.858	2.8181	0.311
Vision (impaired; reference: non impaired)	−4.390	2.7860	0.115	−1.730	1.0865	0.111	−0.623	1.5515	0.688	−1.651	1.3539	0.223	−3.856	5.0079	0.441
Hearing (impaired; reference: non impaired)	1.092	2.4885	0.661	−0.430	0.9706	0.658	0.514	1.3755	0.709	1.280	1.2017	0.287	−2.720	4.4429	0.540
BMI, kg/m^2^	−0.001	0.1381	0.994	0.066	0.0535	0.220	0.125	0.0757	0.098	−0.153	0.0657	*0.020*	−0.388	0.2447	0.113
Waist circumference, cm	0.046	0.0558	0.406	0.028	0.0217	0.199	0.082	0.0298	*0.006*	−0.059	0.0268	*0.029*	−0.050	0.1007	0.618
Medications (1; reference: 2+)	−2.636	2.0071	0.189	0.090	0.7873	0.909	−1.769	1.1062	0.110	−0.823	0.9755	0.399	−1.339	3.6044	0.710
Supplements (0; reference: 1+)	−0.095	1.4198	0.947	−0.367	0.5530	0.507	−0.393	0.7840	0.616	0.121	0.6877	0.860	5.436	2.4976	*0.030*
Alcohol consumption (never; reference: regular)	−2.726	1.8244	0.135	−0.643	0.7194	0.371	−1.759	1.0080	0.081	0.045	0.8956	0.960	−3.688	3.2937	0.263
(Occasional; reference: regular)	1.112	1.5913	0.485	0.185	0.6275	0.768	0.318	0.8793	0.718	0.614	0.7812	0.432	−0.049	2.8729	0.986
Smoking (non-smoker; reference: smoker)	5.788	8.5987	0.501	1.192	3.3576	0.723	5.772	4.7360	0.223	−0.797	4.1710	0.849	−3.793	15.3810	0.805
NEADL score	0.057	0.2766	0.836	0.009	0.1079	0.935	0.006	0.1529	0.969	0.063	0.1339	0.638	−0.207	0.4940	0.676
SPPB score	−0.080	0.3031	0.791	0.049	0.1182	0.681	−0.035	0.1675	0.836	−0.112	0.1466	0.444	0.179	0.5414	0.740
MoCA score (not impaired; reference: impaired)	1.281	1.7996	0.477	0.604	0.6973	0.386	0.868	0.9930	0.382	0.050	0.8717	0.954	−2.413	3.2173	0.453
GDS score (no depression; reference: depression)	0.079	1.8228	0.965	0.909	0.7071	0.199	−0.094	1.0073	0.926	−0.783	0.8807	0.374	0.474	3.2561	0.884

Italicized *p*-values are significant at the 0.05 level.

**Table A3:** Univariate characteristics associated with DQI-I and subcomponents for Plausible reporters only.

Demographic and Health Variables	DQI-I			Variety			Adequacy			Moderation			Balance		
	B	Std. Error	*p*-Value	B	Std. Error	*p*-Value	B	Std. Error	*p*-Value	B	Std. Error	*p*-Value	B	Std. Error	*p*-Value
Age, (<80 years; reference: 80+ years)	−1.252	0.8823	0.156	−0.108	0.3431	0.753	−0.157	0.4212	0.709	−0.761	0.4480	0.090	−2.258	1.6636	0.175
Sex (male; reference: female)	−0.730	0.8991	0.417	0.603	0.3472	0.083	−0.188	0.4282	0.661	−1.190	0.4527	*0.009*	0.450	1.6962	0.791
Ethnic group (European; reference: non-European)	3.720	1.4864	*0.012*	0.680	0.5807	0.242	2.026	0.7053	*0.004*	0.864	0.7618	0.257	1.508	2.8284	0.594
Education level (primary; reference: tertiary)	−0.399	2.5654	0.876	−0.879	0.9930	0.376	−2.029	1.2146	0.095	1.627	1.3014	0.211	8.825	4.8104	0.067
(Secondary; reference: tertiary)	0.228	0.9074	0.802	−0.238	0.3512	0.499	0.190	0.4296	0.659	0.192	0.4603	0.676	0.836	1.7014	0.623
Live with (alone; reference: others)	0.805	0.8860	0.363	−0.106	0.3439	0.757	−0.237	0.4221	0.574	1.182	0.4461	*0.008*	−0.335	1.6721	0.841
Deprivation (low; reference: high)	1.705	1.0818	0.115	0.750	0.4183	0.073	0.658	0.5155	0.202	0.020	0.5522	0.970	2.769	2.0418	0.175
(Medium; reference: high)	1.378	1.1286	0.222	0.821	0.4363	0.060	0.630	0.5378	0.241	−0.238	0.5760	0.680	1.646	2.1300	0.440
Medical conditions (1; reference: 2+)	1.014	1.0485	0.333	0.256	0.4068	0.529	0.208	0.4997	0.677	0.369	0.5336	0.489	1.815	1.9767	0.359
Vision (impaired; reference: non impaired)	1.257	1.7273	0.467	0.031	0.6702	0.963	1.107	0.8205	0.177	0.562	0.8785	0.523	−4.422	3.2490	0.173
Hearing (impaired; reference: non impaired)	2.267	1.7240	0.188	0.040	0.6702	0.953	1.069	0.8206	0.193	1.002	0.8772	0.254	1.581	3.2574	0.627
BMI, kg/m^2^	−0.171	0.1028	0.096	0.000	0.0399	0.991	−0.011	0.0491	0.817	−0.134	0.0520	*0.010*	−0.249	0.1936	0.198
Waist circumference, cm	−0.064	0.0367	0.083	0.013	0.0143	0.345	−0.015	0.0175	0.383	−0.056	0.0185	*0.002*	−0.055	0.0695	0.428
Medications (1; reference: 2+)	0.726	1.6297	0.656	1.286	0.6278	*0.041*	0.122	0.7759	0.875	−0.556	0.8283	0.502	−1.265	3.0719	0.681
Supplements (0; reference: 1+)	0.053	0.8877	0.953	−0.118	0.3441	0.733	−0.017	0.4225	0.969	−0.023	0.4514	0.960	2.096	1.6690	0.209
Alcohol consumption (never; reference: regular)	1.012	1.1664	0.386	−0.817	0.4504	0.070	0.564	0.5548	0.309	0.581	0.5930	0.327	6.840	2.1656	*0.002*
(Occasional; reference: regular)	0.755	1.0085	0.454	−0.405	0.3894	0.298	0.461	0.4797	0.337	0.311	0.5127	0.544	3.883	1.8724	*0.038*
Smoking (non-smoker; reference: smoker)	5.214	3.9235	0.184	2.823	1.5169	0.063	4.061	1.8586	*0.029*	−3.235	1.9923	0.104	15.647	7.3633	*0.034*
NEADL score	0.266	0.2050	0.195	0.038	0.0796	0.632	0.164	0.0974	0.092	0.077	0.1044	0.462	−0.132	0.3873	0.733
SPPB score	0.195	0.2209	0.377	0.135	0.0854	0.114	0.029	0.1052	0.784	0.047	0.1124	0.674	−0.162	0.4167	0.697
MoCA score (not impaired; reference: impaired)	−0.982	1.0486	0.349	−0.505	0.4061	0.213	−0.650	0.4985	0.192	0.777	0.5322	0.144	−6.024	1.9498	*0.002*
GDS score (no depression; reference: depression)	−0.397	1.1694	0.734	−0.683	0.4518	0.131	0.037	0.5567	0.947	−0.016	0.5948	0.979	2.647	2.1996	0.229

Italicized *p*-values are significant at the 0.05 level.
