# Supplementary material for: Sociodemographic and Health Indicators of Diet Quality in Pre-Frail Older Adults in New Zealand
Source: Nutrients. 2023 Oct 18;15(20):4416. doi: 10.3390/nu15204416 (PMC10610025; doi:10.3390/nu15204416)
Supplement: Supplementary file 1 [file nutrients-15-04416-s001.zip › nutrients-2594845-supplementary.pdf]

Article

Sociodemographic and Health Indicators of Diet Quality in Pre-Frail Older Adults in New Zealand

Esther Tay [1], Daniel Barnett [2], Maisie Rowland [3], Ngaire Kerse [1], Richard Edlin [4], Debra L. Waters [5,6,7], Martin Connolly [8], Avinesh Pillai [2], Evelingi Tupou [1] and Ruth Teh [1,*]

Supplementary Material

Materials and Methods

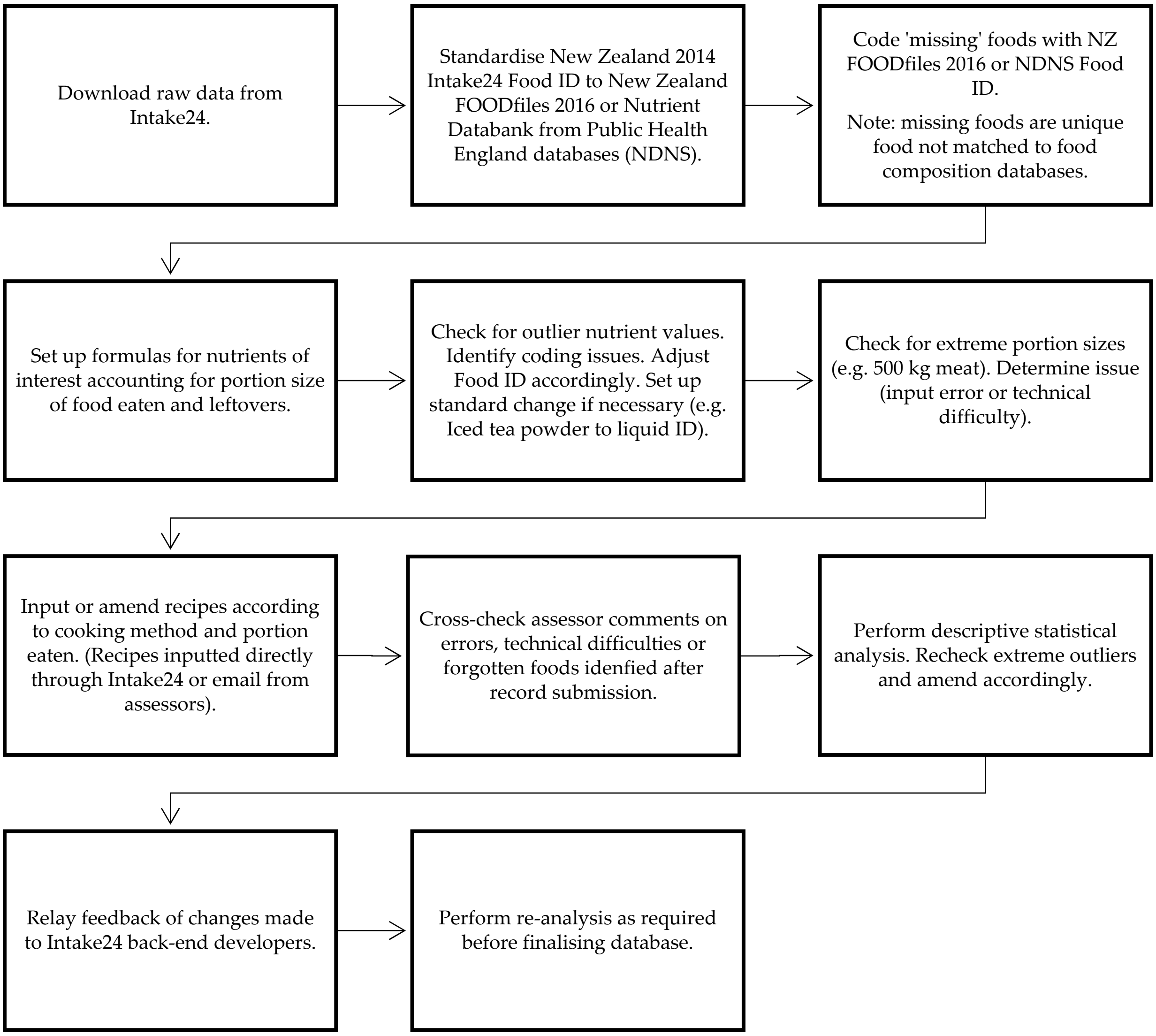

Figure S1 Summary of systems and changes implemented for cleaning Intake24 data.

Table S1. Multivariate regression against DQI-I score and subcomponents for Low Energy Reporter (LER) only.

Demographic and health variables	B	95% Wald CI	*p*-value
**DQI-I total score**			
(Intercept)	58.567	52.248, 64.887	*<0.001*
Live alone; (reference: with others)	2.480	-0.300, 5.260	0.080
Alcohol consumption (never; reference: regular)	-2.382	-5.921, 1.156	0.187
(Occasional; reference: regular)	0.680	-2.404, 3.764	0.666
Vision (impaired; reference: non impaired)	-3.890	-9.254, 1.474	0.155
Deprivation (low; reference: high)	2.436	-1.027, 5.898	0.168
(Medium; reference: high)	2.125	-1.208, 5.457	0.211
(Scale)	68.016	54.281, 85.226	
**Variety score**			
(Intercept)	7.743	3.259, 12.227	*<0.001*
Live alone; (reference: with others)	0.987	-0.086, 2.059	0.071
Waist circumference, cm	0.027	-0.015, 0.069	0.207
GDS score (no depression; reference: depression)	0.894	-0.473, 2.261	0.200
(Scale)	10.709	8.533, 13.438	
**Adequacy score**			
(Intercept)	16.864	10.581, 23.147	*<0.001*
Deprivation (low; reference: high)	1.499	-0.344, 3.341	0.111
(Medium; reference: high)	2.489	0.689, 4.289	*0.007*
Waist circumference, cm	0.083	0.025, 0.141	*0.005*
Alcohol consumption (never; reference: regular)	-0.920	-2.854, 1.014	0.351
(Occasional; reference: regular)	0.022	-1.626, 1.669	0.979
(Scale)	19.614	15.630, 24.615	
**Moderation score**			
(Intercept)	20.251	16.143, 24.359	*<0.001*
Sex (male; reference: female)	-1.777	-3.135, -0.419	*0.010*
BMI, kg/m^2	-0.177	-0.303, -0.051	*0.006*
Live alone; (reference: with others)	0.602	-0.762, 1.966	0.387
(Scale)	15.798	12.598, 19.810	
**Balance score**			
(Intercept)	5.391	3.779, 7.004	*<0.001*
Supplements (0; reference: 1+)	0.514	0.028, 1.000	*0.038*
BMI, kg/m^2	-0.031	-0.078, 0.016	0.189
Sex (male; reference: female)	0.401	-0.087, 0.889	0.107
Education level (primary; reference: tertiary)	-0.989	-2.500, 0.522	0.199
(Secondary; reference: tertiary)	-0.097	-0.584, 0.391	0.698
(Scale)	2.188	1.745, 2.744	

Italicized *p*-values are significant at the 0.05 level.

Table S2. Multivariate regression against DQI-I score and subcomponents for plausible reporters only.

Demographic and health variables	B	95% Wald CI	*p*-value
**DQI-I total score**			
(Intercept)	59.995	51.618, 68.372	*<0.001*
Ethnic group (European; reference: non-European)	3.034	0.016, 6.052	*0.049*
Waist circumference, cm	-.039	-0.113, 0.035	0.299
Live alone; (reference: with others)	0.807	-0.936, 2.550	0.364
Deprivation (low; reference: high)	1.443	-0.695, 3.581	0.186
(Medium; reference: high)	0.893	-1.330, 3.116	0.431
(Scale)	59.223	50.634, 69.267	
**Variety score**			
(Intercept)	13.526	12.693, 14.360	*<0.001*
Medications (1; reference: 2+)	1.191	-0.024, 2.406	0.055
Sex (male; reference: female)	0.491	-0.192, 1.173	0.159
Deprivation (low; reference: high)	0.624	-0.190, 1.437	0.133
(Medium; reference: high)	0.751	-0.094, 1.596	0.082
Alcohol consumption (never; reference: regular)	-0.682	-1.557, 0.193	0.127
(Occasional; reference: regular)	-0.278	-1.041, 0.486	0.476
(Scale)	8.812	7.534, 10.306	
**Adequacy score**			
(Intercept)	21.257	16.126, 26.387	*<0.001*
Ethnic group (European; reference: non-European)	2.088	0.724, 3.453	*0.003*
Smoking (non-smoker; reference: smoker)	4.062	0.457, 7.666	*0.027*
Education level (primary; reference: tertiary)	-1.833	-4.165, 0.499	0.123
(Secondary; reference: tertiary)	0.079	-0.750, 0.908	0.851
NEADL score	0.161	-0.026, 0.348	0.091
(Scale)	13.025	11.136, 16.234	
**Moderation score**			
(Intercept)	14.275	8.80, 19.744	*<0.001*
Live alone; (reference: with others)	0.927	0.031, 1.823	*0.043*
Age, (<80 years; reference: 80+ years)	-0.452	-1.356, 0.452	0.327
MoCA score (not impaired; reference: impaired)	0.778	-0.244, 1.799	0.136
Waist circumference, cm	-0.048	-0.084, -0.012	*0.010*
NEADL score	0.047	-0.156, 0.250	0.647
(Scale)	14.938	12.771, 17.471	
**Balance score**			
(Intercept)	4.652	4.259, 5.046	*<0.001*
Alcohol consumption (never; reference: regular)	0.674	0.256, 1.092	*0.002*
(Occasional; reference: regular)	0.373	0.011, 0.734	*0.043*
MoCA score (not impaired; reference: impaired)	-0.590	-0.966, -0.214	*0.002*
(Scale)	2.040	1.744, 2.386	

Italicized *p*-values are significant at the 0.05 level.

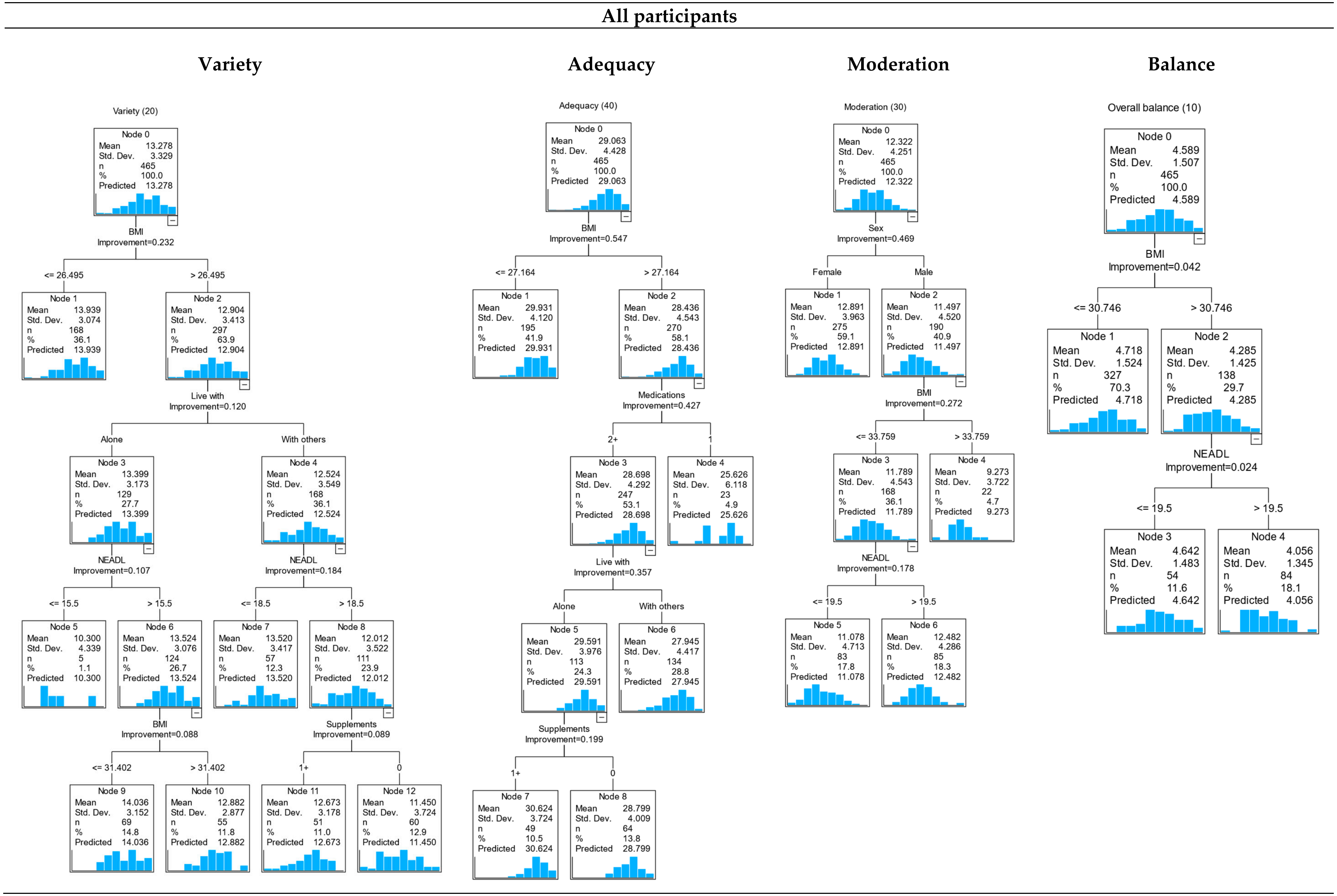

Figure S2. CART diagram of factors associated with DQI-I subcomponents in pre-frail older adults

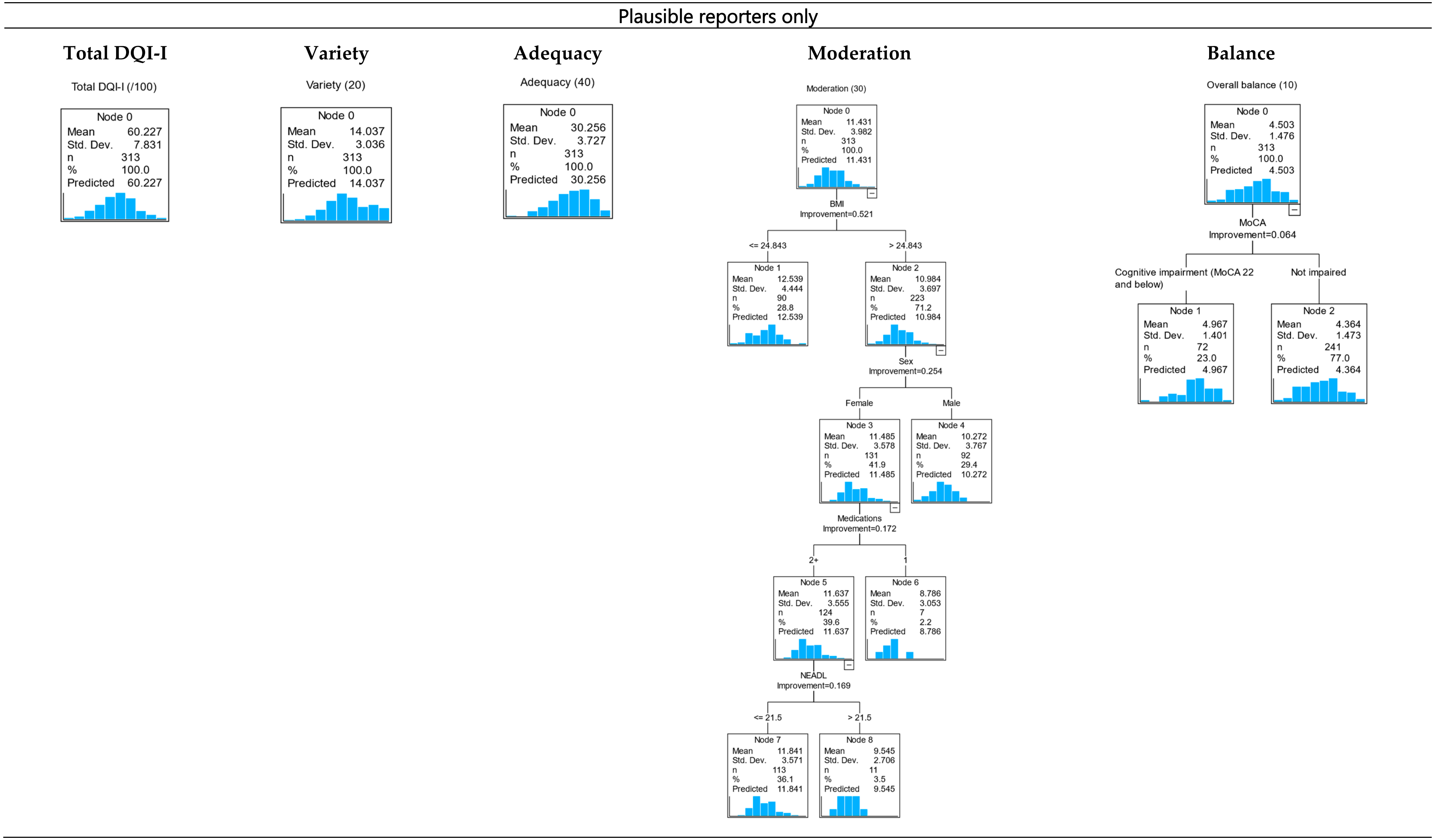

Figure S3. CART diagram of factors associated with DQI-I and subcomponents in pre-frail older adults for plausible reporter (PR) only.

Low Energy Reporters only

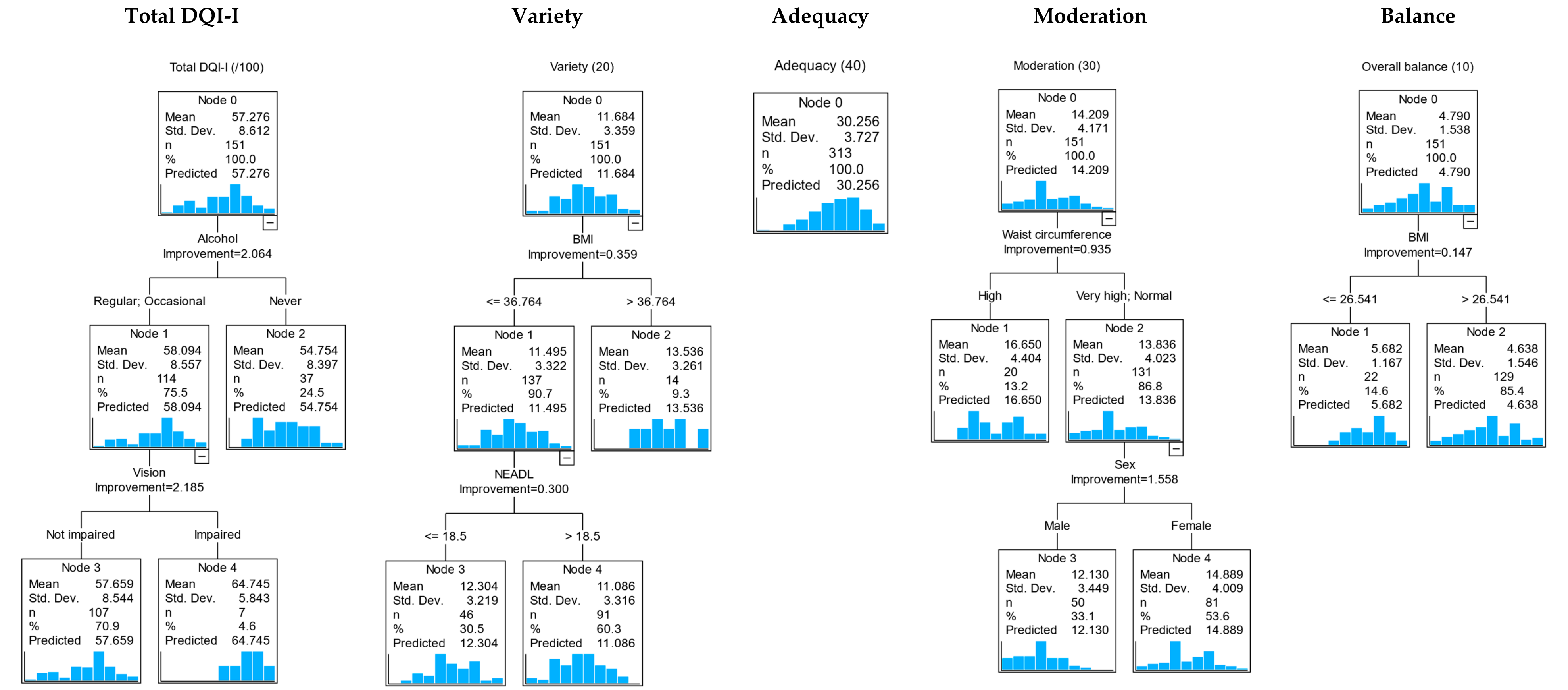

Figure S4. CART diagram of factors associated with DQI-I and subcomponents in pre-frail older adults for LER only.
